# Managers in the context of small business growth: a qualitative study of working conditions and wellbeing

**DOI:** 10.1186/s12889-024-19578-4

**Published:** 2024-07-31

**Authors:** Elena Ahmadi, Daniel Lundqvist, Gunnar Bergström, Gloria Macassa

**Affiliations:** 1 Department of Occupational Health, Psychology and Sports Sciences, Faculty of Health and Occupational Studies, University of Gävle, Gävle, 80176 Sweden; 2 Department of Business and Economic Studies, Faculty of Education and Business Studies, University of Gävle, Gävle, Sweden; 3 Department of Behavioural Sciences and Learning, Division of Education and Sociology, Linköping University, Linköping, Sweden; 4 Unit of Intervention and Implementation Research for Worker Health, Institute of Environmental Medicine, Karolinska Institute, Box 210, Stockholm, 171 77 Sweden; 5 Department of Social Work, Criminology and Public Health Sciences, Faculty of Health and Occupational Studies, University of Gävle, Gävle, Sweden; 6 EPIUnit–Instituto de Saude Publica, Universidade do Porto, Porto, Portugal

**Keywords:** Small businesses, Business growth, Managers, Wellbeing, Psychosocial working conditions, Job demands, Job resources

## Abstract

**Purpose:**

In view of the importance of managers’ wellbeing for their leadership behaviour, employee health, and business effectiveness and survival, a better understanding of managers’ wellbeing and working conditions is important for creating healthy and sustainable businesses. Previous research has mostly provided a static picture of managers’ wellbeing and work in the context of small businesses, missing the variability and dynamism that is characteristic of this context. Therefore, the purpose of this study is to explore how managers in small companies perceive their working conditions and wellbeing in the context of business growth.

**Methods:**

The study is based on qualitative semi-structured interviews with 20 managers from twelve small companies. Content and thematic analysis were applied.

**Results:**

The findings indicate that a manager’s working environment evolves from its initial stages and through the company’s growth, leading to variations over time in the manager’s experiences of wellbeing and work–life balance as well as changes in job demands and resources. Managers’ working situation becomes less demanding and more manageable when workloads and working hours are reduced and a better work–life balance is achieved. The perceived improvement is related to changes in organizational factors (e.g. company resources), but also to individual factors (e.g. managers’ increased awareness of the importance of a sustainable work situation). However, there were differences in how the working conditions and wellbeing changed over time and how organizational and individual resources affected the studied managers’ wellbeing.

**Conclusions:**

This study shows that, in the context of small business, managers’ working conditions and wellbeing are dynamic and are linked to growth-related changes that occur from the start of organizational activities and during periods of growth. In addition, the findings suggest that changes in managers’ working conditions and wellbeing follow different trajectories over time because of the interaction between organizational and personal factors.

## Introduction

Small businesses play a significant role in global economies [1, 2] and growing businesses are especially important in creating jobs and contributing to economic growth [3–5]. Previous research has shown the importance of managers’ wellbeing for leadership behaviours [6], employee health [7] and business survival and effectiveness [8, 9]. Managers’ working conditions influence their wellbeing, which is important for their practiced leadership [6, 10], which in its turn has effect on employees’ wellbeing [7, 11–13]. Therefore, a better understanding of managers’ wellbeing and working conditions in the context of small businesses is important for creating healthy and sustainable businesses. Yet too few studies have focused on managers’ health and working conditions in the context of small companies.

Previous research has provided a largely static picture of wellbeing and work in small businesses, missing the variability and dynamism characteristic of this context [14]. One aspect of the dynamic context of small businesses is growth and the changes that growth causes in the companies. Hessels et al. [9] report that increasing firm age and size may have implications for managers’ working situation and wellbeing. More research is needed to examine how business growth can impact managers’ working conditions and wellbeing as this has implications for employees’ wellbeing and company performance. The purpose of this study is to explore perceived changes in working conditions and wellbeing among managers in the context of growing small businesses.

Prior to discussing the methodology of the study, the section below provides a short exposé over the conceptual and theoretical framework of this study as well as an overview of the previous research.

### Theoretical framework and previous research

Through the lens of the Job Demands–Resources (JD–R) model [15–17] this paper explores the wellbeing and working conditions of managers of small businesses. The JD–R model, differentiates between two types of factors in the work environment: job demands and job resources [17]. The term “job demands” refers to job characteristics and circumstances requiring physical and psychological efforts and having physiological and psychological costs [17], e.g. workload and work pace. Resources, e.g. control, autonomy and support, on the other hand, contribute to achieving work goals, personal growth and development, and counterbalance the job demands and related physiological and psychological costs [18]. It has been suggested that working conditions characterized by high demands and low resources lead to increased strain and decreased work engagement [18] while work situations with high job demands and high resources are regarded as active and stimulating [19]. Research has shown that wellbeing, in general, is positively influenced by high job resources and negatively by increased job demands [20–22].

The JD-R model is among the most influential in connecting working conditions to well-being beside the demand–control–support model [23, 24] and the effort–reward imbalance model [25, 26]. This model was chosen since it is flexible, enabling among others the inclusion of working conditions and factors relevant to specific occupational settings [27]. Moreover, it has received empirical support across various contexts [28–31].

Since well-being encompasses several dimensions [32, 33], such as physical, emotional, mental, and social [34, 35], there have emerged quite a few ways of defining this concept. This paper adopts a broad conceptualization of well-being to capture the dimensions and reflecting managers’ evaluation of their lives, feeling well and daily functioning based on their unique perspectives [36–38]. This concept includes individuals’ subjective judgement of their life, work, health, relationships, and sense of purpose, including both positive (such as feeling of job satisfaction and happiness) and negative (such as feelings of distress, health problems impairing individuals’ daily functioning and quality of life) aspects of wellbeing [39].

There have been two strands of research applied to small business, one focusing on the general population of managers and the other focusing on entrepreneurs. However, among these studies, very few to date have specifically addressed managers’ wellbeing and working conditions in the context of small and growing companies. For instance, the published research has not sufficiently distinguished between different types of entrepreneurs, i.e. those with and those without employees [9, 14]. There are differences in the nature of managerial work between that of small and large companies; and there are differences even between smaller firms depending on their size [40]. In addition, in small businesses, manager–owners have the combined responsibilities of entrepreneurs, managers, and operative employees, which may impact their work and wellbeing.

Research shows that both entrepreneurs and managers in general experience stressful working situations and high levels of demands, in terms of long working hours, a high workload and fast work pace, poor work–life balance, role conflicts and low support [8, 14, 41–46]. Entrepreneurs also work under uncertainty and amidst financial problems [8, 14]. On the other hand, both managers and entrepreneurs experience high levels of control, autonomy and decision latitude [14, 41, 47]. Entrepreneurs enjoy flexibility and meaningfulness in work and report high job satisfaction and optimism [8, 14].

Despite the intense demands and stressful work, managers and entrepreneurs generally report good wellbeing, better wellbeing compared with employees and other non-managers [9, 14, 19, 48]. However, several studies have pointed to risks of decreased wellbeing associated with managerial position [49–52]. Similarly, entrepreneurs may run high risk of burnout [8] and ill health in the long term because of continuous exposure to high levels of stressors [14]. A few studies have reported entrepreneurs’ poor wellbeing [53, 54].

Research also highlights differences in the wellbeing of managers based on their hierarchical level, where top managers enjoy better wellbeing, and first-line managers experience worse wellbeing and working conditions [55, 56]. Buttner [4] suggests that entrepreneurs experience more problems with wellbeing, as well as higher stress and lower job satisfaction compared with managers and points to differences in entrepreneurial and managerial work demands.

Regarding business growth, this is known to be a complex and multifaceted phenomenon [57]. Despite a large volume of research, the area still suffers from insufficient theoretical development and a limited understanding [58, 59]. In business studies, one of the approaches to describing business growth can be found in a rich plethora of life cycle models illustrating growth trajectories of firms as passing through a number of stages [60, 61]. However, although the life cycle approach has been challenged for its determinism and linearity [61], researchers agree on the common features in the growth process, which include a series of stable periods, accompanied by crises points, as well as changes in the companies’ basic structure, activities and key challenges over time. In other words, when companies grow there are certain transformations beyond change in size and age.

According to a model by Churchill and Lewis [62], which was specifically developed for small growing companies, businesses move through five growth stages, namely, existence, survival, take-off, success, and resource maturity. Each stage involves an increase in diversity and complexity of five management factors: managerial style and management decision making (including the extent to which decision making authority is delegated by the owner); organizational structure (involving layers of management in the company); operational systems (referring to the development of financial, marketing, and production systems in the company); strategic planning (the degree to which a company develops both short- and long-range goals as well as major strategic planning); and owner involvement (the extent to which the owner is active in the business operations and decisions). The set of core problems and challenges that managers face also changes through the stages [62, 63]. According to Churchill and Lewis [62], as a business moves through the growth stages, the owner’s style of decision making changes and becomes less controlling and more delegating. This means that owner involvement in the firm and daily work decreases and a new layer of management is created, with new managers coming in, as well as there being an increase in the complexity of organizational structure, operational systems, and strategic planning.

Torrès and Julien’s [64] discussion on the denaturing of small businesses (i.e. when the businesses no longer have the typical features of small businesses and adopt attributes that belong to larger companies) can help understand change related to growth. According to Torrrès and Julien [64], the denaturing of small business management practices can be marked by a higher degree of management decentralization, higher levels of labour specialization, the development of more formal, long-term strategies, a growing complexity, and formalization of information systems, as well as expanded markets. Denaturing is also followed by decreasing proximity in relations and contacts, growing formality and procedures, and a more structured and long-term approach [64].

Thus, growth (in size and complexity) introduces changes in a company’s structural and contextual dimension [61] that have consequences for the nature of the manager’s role [63] and, supposedly, for managers’ working conditions, resources and demands. However, little attention has been paid to business growth from an occupational health perspective.

Therefore, as stated above, this study explores how managers in small companies perceive their working conditions and wellbeing in the context of business growth. Following the theoretical foundation, the next section sets the stage for discussing the methodology that was used in this study.

## Materials and methods

### Study design and sample

This study used a qualitative methodology based on interviews with managers of small companies. The company selection was linked to a regional project, Successful Companies in Gästrikland (SCiG), which annually credits successful businesses (ranked highest in terms of profitable growth) in a region in mid-Sweden. The selection procedure is fully described elsewhere [40].

For this study, we selected small companies (max 50 employees) that were nominated for the award between 2008 and 2019 and had been in operation since 2008 at least. Interviews were performed with 20 managers from twelve companies. The heterogeneity of the sample was increased by purposefully selecting companies on the top and at the bottom of the nomination list for the period 2008–2019. Nine companies had more than seven nominations during the period (indicating sustained profitable growth); three companies had only one nomination (indicating a short growth period).

The chief executive officers (CEOs) of the selected companies were invited by letter and subsequent phone calls to participate in the study. They were provided with information about the study’s purpose, methodology, and treatment of the collected data. The companies were in sales (*n* = 5), manufacturing (*n* = 4), technical consultancy (*n* = 2), and transportation (*n* = 1), employed between four and 46 persons and had been in operation for 12–51 years.

Twelve CEOs, nine of whom were owner–managers, and eight managers at lower level made up the group of participants. Managers of different levels were included to increase the variation in the material as the situation of low-level managers can differ from that of top managers. The participants included 18 male and two female managers between the ages of 29 and 66. Their managerial experience ranged from 3 to 29 years. Four managers were university-educated; 16 had secondary education or similar. Table 1 illustrates an overview of the characteristics of the managers participating in the study.

**Table 1:** Characteristics of the participants

Interview Person	Position	Gender	Manager experience	Tenure	Industry	Company size
IP1	CEO, owner	Male	29	29	Sales	14
IP2	Lower manager	Male	15	2	Sales	14
IP3	CEO, owner	Male	26	12	Sales	13
IP4	CEO, owner	Male	17	17	Manufacturing	40
IP5	Lower manager	Male	15	1	Manufacturing	40
IP6	CEO, owner	Male	17	17	Manufacturing	17
IP7	Lower manager	Male	4	13	Manufacturing	17
IP8	CEO, owner	Male	13	13	Manufacturing	29
IP9	Lower manager	Male	7	25	Manufacturing	29
IP10	CEO	Female	20	1	Sales	10
IP11	CEO, owner	Male	15	18	Technical consultancy	28
IP12	Lower manager	Male	6	14	Technical consultancy	28
IP13	CEO, owner	Male	15	20	Transportation	46
IP14	Lower manager	Male	4	2	Transportation	46
IP15	CEO	Male	27	27	Sales	17
IP16	Lower manager	Female	8	4	Sales	17
IP17	CEO, owner	Male	16	16	Technical consultancy	4
IP18	CEO, owner	Male	3	14	Sales	6
IP19	Lower manager	Male	6	14	Manufacturing	6
IP20	CEO, owner	Male	26	26	Manufacturing	6

### Data collection

The qualitative interviews were performed in 2020. A semi-structured interview guide [65] was employed and included such themes as experiences of managers’ wellbeing, working conditions, and work-related factors influencing their wellbeing. Examples of questions were: “How do you perceive your own health and wellbeing?”, “Did your wellbeing change during your work as manager? – If yes, in what way, and what did it depend on?”, “How do you perceive your work–life balance?” and “How do you perceive your working situation?” The open-ended questions were followed by probing questions to follow up and get clarifications and examples. This procedure enabled a natural conversation where interviewees could freely describe their perspectives. The participants were not provided with a definition of “wellbeing”.

The interviews were carried out by the first author either at the companies (*n* = 18) or remotely using the video conferencing service Zoom (*n* = 2). The interviews lasted 60–90 min. With the participants’ permission, all interviews were audio-recorded. A professional transcriber (*n* = 17) and the first author (*n* = 3) transcribed the interviews verbatim.

### Data analysis

Data analysis was performed in two complementary stages. In the first stage, the data were analysed using qualitative content analysis [66–68]. Following the guidelines of Elo & Kyngäs [66] and Graneheim & Lundman [67] the content analysis followed such steps as preparation (selecting the unit of analysis and familiarizing with the data), organization (open coding, grouping, categorization, and abstraction) and reporting. The interview transcripts in their entirety were regarded as units of analysis [67]. They were read several times to achieve immersion in the data. The texts were thereafter uploaded to ATLAS.ti for Windows, version 9 (Microsoft, Seattle, WA, USA) for subsequent analysis.

All information in the interview transcripts, that was judged as relevant for the objective of the study, was thoroughly coded. The coding was done by selecting meaning units (ranging from a few words to several sentences) and assigning a heading that reflected their meaning and content. These headings became the initial codes. For instance, the phrase “My health was quite poor. Poor sleep. … Notepad on the bedside table so when you woke up at night and thought of things you had to write them down…. It wears you out a lot. You get old, you know, inside you age quickly… (IP1)” was coded as “Felt unwell previously”.

These initial codes were then compared with each other for similarities and differences, sorted, and abstracted into broader categories. The coding scheme was revised and refined several times through the iterative processes of sorting and abstraction, comparing meaning units, codes, categories, and subcategories. Thus, the content analysis in the first stage resulted in a list of categories, subcategories and codes describing managers’ perception of changes in their wellbeing and working conditions. These are presented in a category matrix (Table 2), elaborated, and supported by participants’ quotes in the Results section.

**Table 2:** Categories of change in the managers’ wellbeing and working conditions

Categories and subcategories		Codes
1. Wellbeing and work–life balance		
	1.1. Currently	Feel well generally Would like to exercise more Rarely stressed, or stressed for only shorter periods Satisfied with the job; thrive at work Good balance between work and private life
	1.2. Previously	Felt unwell, stressed More stressed previously because of working long hours and not having time for family and private life Poor work–life balance in previous periods Still satisfied with job despite high workload
2. Demands		
	2.1. Currently	Experience high but manageable workload now Working hours of 40–60 h/week New challenges due to growth: • need to work with adjustments in the organization to match the growing size and the deepening complexity of the company • problems in the organization, unclear structure, roles, lack of policies, routines, uncertainty, conflicts, staff turnover, difficulties to maintain family climate and close relationships
	2.2. Previously	High workload and excessive working hours of 50–100 h/week; worked evenings and holidays Low control over time and work Negative consequences for wellbeing and life situation
3. Resources		
	3.1. Change in organizational resources	Increased financial, personnel and organizational resources for companies with continuous growth, with clear implications for managers’ work
	3.2. Change in individual resources	Awareness of the importance of wellbeing and a sustainable working situation Intentional adjustments in the working situation; prioritizing health and striving for better work–family balance and a sustainable work situation Better in coping, work experience, personality

In the second stage, thematic analysis was employed to identify trajectories in the participants’ perceptions of their wellbeing, demands and resources (which are the categories identified in the first stage of analysis). This was done based on their descriptions of their working situation, currently and previously, as manager of the business. All the transcripts were reread several times and individual trajectories of the perceived changes in the factors were summarized for each case. These individual trajectories were aggregated in groups showing commonalities and differences in the participants’ experiences in how their perceived wellbeing, demands and resources had changed from previous periods to the time of data collection. The pattern grouping showed more salient trajectories where individuals could be a part of several groups. As a result, the analysis in the second stage suggested themes illustrating common patterns in participants’ individual trajectories of perceived wellbeing, demands and resources.

The main analysis was done by the first author (E.A.). Sorting and abstraction of data was then discussed with the second author (D.L.). Finally, the categories and themes were reviewed by all authors. The analysis presented in the results section is performed close to the manifest content [67] reflecting the perceptions and experiences of managers as expressed by them, and with low degree of interpretation by the authors. Further interpretation, analysis of connections between the categories and theorizing is done in the discussion section.

### Ethical considerations

The study was approved by the Swedish Ethical Review Authority (approval No. 2019 − 00314). Furthermore, the study was carried out in line with the principles of the Declaration of Helsinki. All participants were informed about the study’s objective, the voluntariness of participation, anonymity, and confidentiality principles as well as their right to decline an interview at any moment without having to provide a justification. Before each interview, informed consent was gained from each participant.

Following the discussion of the methodology, the next section offers the reader an overview of the results of the empirical study.

## Results

The results are presented in two sections corresponding to the two stages of the analysis. In the first section we present findings showing that there has been change in managers’ experience of their wellbeing and working conditions from previously to today, and what factors were affected (see Table 2). The second section presents the findings of a trajectory analysis, individually investigating each manager’s journey and illustrating different ways in which these changes occurred.

### Categories describing changes in the managers’ wellbeing and working conditions

#### a. Managers’ wellbeing and work-life balance currently and previously

The managers stated that they were satisfied with their job, and that they thrived at work. Several participants maintained that it was fun to go to work and that work gave them energy. Most of the managers assessed themselves as feeling well. Some said that their physical health could be better, e.g. they referred to problems of overweight or problems due to having a prolonged sitting time at work.

I feel great in many ways. Physically, it’s so-so considering that I’m overweight! Occupational health is great when I don’t work 100% and I’m in charge of my own free time. (Interview participant (IP) 1, CEO)

Certainly it has been up and down, but I perceive my health as good. It makes me feel good when I come here [to work]; I enjoy it a lot. And that gives me a lot of energy. (IP 16, lower manager)

Several managers expressed that they were down-prioritizing their physical health in favour of spending time with family and of doing managerial work. Some maintained that they had not done what they should have done for their health to be better. Several participants wished that they could do more exercise.

Managers referred to feeling stressed during certain periods because of high workload and work pace; however, the stress was not constant. They described that it “goes in waves” and that work had “ups and downs.” Most managers stated that they were rarely badly stressed and when they were, it was for shorter time periods. Also, the managers felt they had a good balance between work and private life currently.

Several owner–managers expressed that the sheer fact that they had the opportunity to carry out what made them thrive compensated for the heavy burden of having to work long hours. Some noted that they felt calm when there was a lot of work and a high tempo because it meant that the company had a lot of orders and it was going well for the business.

You enjoy your job, but you may work a little more, but you get a good life situation yourself. … I like to work … I like to have many “irons in the fire”. That’s when I’m at my calmest … as long as it’s full speed and challenges like that … (IP 4, CEO).

Several managers, however, stated that they had felt unwell in earlier periods of their managerial career. They reported having felt tired, worn out and stressed constantly over longer periods of time. Some felt that they had prioritized away their health, had not had time to take care of themselves, to have lunch or take breaks, and had overconsumed coffee and tobacco. Several participants had since had problems with physical and mental health, including problems with the heart and stomach, burnout, and stress-induced shingles.

From the beginning … when I started … I worked very, very hard and then my stomach took a beating … and I’ve always been a bit stingy [which is why this CEO did not hire staff to do some of the work]. So I worked evenings and nights … until 3 in the morning … well, I probably did it for about 5 years …. So that’s when I realized I can’t go on like this. (IP 3, CEO)

The first year I got gastritis and I started to feel dizzy, because I worked extremely hard. I went to the doctor and I used a pack of snus a day, drank twelve cups of coffee a day. I guess I didn’t realize my situation, that I have a big family plus a job. (IP 19, CEO)

Several managers stated that it had been a problem when they had worked more in the past. They described that a high workload and long working hours made it difficult to combine running a business and having a family life. They discussed that it is usual for small business owners to have difficulties with a relationship and family because of a large workload. Many managers felt that, previously, they had had no control over their own time and no time for family or relationships.

It’s hard to have your own business. But … if you have your own business and you feel that you’re managing your free time, then you’ve come to the right place. However, if you have to work 100 h a week because you have a bad conscience about things, then you’re not in control of your own time. You won’t exactly be a nice person. Because you’re never at home, you’re never free … It doesn’t work with a relationship … from a family’s point of view, it’s probably really hard to be together with a self-employed person. (IP 1, CEO )

The man I bought the company from … ran it for 4 years; then his wife said, You have to choose between me and the job. So then I was alone [in running the business]. I didn’t have a holiday. I had two small children, I worked every day of the week, between 7am and 9pm every day except weekends when I worked a little less but basically I worked all the time. Then my wife said, Now you have to choose, between the company and the family. (IP 8, CEO)

#### b. Demands currently and previously

Most of the managers described their current workload as quite high, but manageable. They estimated that they worked between 40 and 60 h a week, and generally did not see that as problematic. They knew the workload went up and down, in waves, and intense periods were followed by calmer periods during which they could recover. Some managers when talking about small business owners in general said that it is inevitable to work long hours – it is a common situation. They described that, when running an own business, one can never feel that the work is finished as there is always something to solve or improve.

When you run a business, you’re never done. There are always improvements to be made. You can never sit down and feel that now things are good. We want to improve our production, routines. At the same time, the most important thing is to be able to deliver, both products and services. That’s what we live on, so to speak. If we can’t do that, we don’t make any money. Then we’ll be out of business soon. (IP 20, CEO)

Some managers also reported that company growth brought about new challenges for them to handle. They expressed that there was a constant need for adjustments in the organization to match the growing size and complexity of the tasks performed by the company. In addition, some participants talked about the challenges in the organization related to clarity of structure, roles, policies, routines and information as companies expanded. Some managers mentioned conflicts and staff turnover in some periods as well as difficulties to maintain the family climate and close relationships.

Talking about previous periods of their managerial career in the current company, many managers described having worked much more compared with their present work situation. They estimated having worked between 50 and 100 h a week, including evenings and holidays.

Before, I worked a lot more. Maybe 100 h a week. I have done that for many years. Probably 20 years I would think … Lots of night work. Came home at 2, 3 and then up again at … (IP 1, CEO).

At the same time, the managers said that it was fun and they enjoyed working in this period of heavy workload and long working hours. Several managers explained that they were very engaged and ambitious, and wanted to achieve much more. Some described that they just worked and worked. One mentioned that he kept going as if he was a superman, another as if she was immortal; both meant that they felt they could manage anything and did not realize their limits.

I think you have a great overconfidence in yourself; in the beginning you want to do everything, you want to change yourself, you want to change the company, you have made an investment. Then after a while you realize that life is more than just work, life is more than money. (IP 8, CEO)

#### c. Resources

**c1. Change in organizational resources**

In the managers’ descriptions of their current and previous working conditions, they often referred to changes in the available organizational resources due to the growth of the company. They described that previously, when the company had been small, they had had multiple roles and had done almost everything in the company, operative work, administration, and management. All activities in the business had been theirs. They had felt they needed to be present all the time to ensure that everything ran smoothly and had done as much as they could themselves to save costs and build a stronger financial base for the company.

When the company had expanded, they had acquired financial and personnel resources. A new group of managers (at a lower level) had been hired, who had taken over some of the responsibility for staff and daily operative leadership. Extra staff had been hired to take care of finance and administration, relieving the managers from these tasks.

The acquisition of additional job resources was particularly prominent in CEOs’ perceptions of wellbeing. They felt that their workload decreased as they could delegate responsibility and tasks to lower-level managers, technical–administrative staff, and other employees. The CEOs could work more purposefully on overall leadership, and more proactively with development and seeking new clients, which, in their eyes, meant a purer leadership role, focusing on managerial tasks.

A few years ago, you were more of a salesperson and then you would get into a new suit and then you would go in and manage people. It was completely new … You do not do everything, you do [only] your thing. (IP 1, CEO)

I had more to do and then it simply took longer. Now I have less to do. My work tasks are now shared by more people. (IP 4, CEO)

Some companies assigned both lower-level managers and other staff to take care of improvements in certain key areas, such as optimization of organization and processes, the work environment and safety, quality, certification, documenting routines, etc. The CEOs reported that they had not had the time to take care of these issues before. Finally, the process of growth required changes in the organization. The managers described that when the company expanded, there was an increase in specialization and division into departments or groups. The companies developed a clearer organization, roles and routines, which, according to the managers, contributed to a smoother processes and more effective problem solving.

We’ve made a lot of changes over the years, from chaos to organized chaos to order. Now that we have an organization, I work much less. (IP 1, CEO)

While most companies in the study showed continuous growth, three companies did not. The managers of these companies did not mention gaining organizational resources, but instead described the vulnerability of being a small business that was related to lack of financial and personnel resources.

**c2. Change in individual resources**

Many managers said that they had come to the insight that their work situation was not sustainable in the long run and needed to be changed.

To have that pace forever, then you give up in the end … but if you enjoy it and want to continue working then you have to try to find a sustainable work situation that works both at work and at home. … because otherwise you end up as a human being that you won’t be able to bear. You have nothing more to give … and it’s certain that you will burn yourself and others out. (IP 16, lower manager)

Two factors had led to this insight: ill health and the family situation. Some managers realized the importance of wellbeing and a sustainable work situation after having problems with their health and work–life balance. Those who developed health problems described how this had become a strong warning signal.

I got burned out 10 years ago. … And there it stopped. So I learned then. It was absolutely the most useful lesson I could have received. (IP 13, CEO)

Several managers expressed that they now prioritized health more and strove for a better work–life balance and a more sustainable work situation. Some worked intently on changing their situation and reducing their own working time. Some also maintained that they kept the balance over a long period of time, meaning that they worked overtime some days but compensated for it by working less on other days.

Some managers also mentioned a change in their family situation and their relationship with their partner and children as factors that had made them aware of the importance of wellbeing and work–life balance and had convinced them to make changes in the working situation. One participant talked about age as playing a role in this context. He emphasized that now, closer to retirement age, he did not want to work too much. He wanted more free time.

I work less now. I worked a lot more in the past! I don’t want to work as much. I’ve handed over things like administration, preparation of orders, and so on to the deputy manager. (IP 20, CEO)

Several managers specially highlighted the importance of accumulated managerial experience. They described that they had become more secure in their role and had reached a better understanding of the situation and the yearly work cycle. Based on this they made quicker decisions and did not spend so much time on seeking information. They also described that they had learned to cope better with the work situation, e.g. through planning, prioritizing, working in a more structured way, accomplishing work bit by bit, not promising too much and accepting that stress and a high workload are part of a manager’s job.

### Trajectories of managers’ wellbeing and work conditions in the context of the growth of small businesses

The trajectory analysis showed that the changes in the managers’ wellbeing and working conditions occurred in different ways. Despite large variation in experiences, individual and firm-level characteristics and circumstances, several groups of trajectories of participants’ wellbeing and working conditions were identified.

#### a. Changes in wellbeing due to organizational and individual resources

This group consisted of owner–managers of growing companies who experienced changes in wellbeing as well as in organizational and individual factors. The managers in this group reported that, initially, when their companies had been smaller, they had experienced a deterioration in wellbeing because of a high workload, fast work pace and long working hours. However, enhanced organizational and individual resources had led to an improved work situation and wellbeing for this group. As companies had grown, managers had been able to hire more staff who could relieve them or take over some of their tasks. According to the managers in this group, their wellbeing had improved over time, from having been stressed to a new experience of feeling good. Challenges to wellbeing and disruption of the work–life balance had provided them with increased awareness of the importance of a sustainable working life and their own wellbeing. Several managers described that they had specifically worked on changing both their own work environment and the organization to make the company less dependent on the owner–manager’s availability all the time.

#### b. Unchanged wellbeing

The managers in this group had a stable wellbeing and had not experienced any significant changes in their wellbeing due to their work. Some managers noted that owing to their coping strategies (positive personality and taking things as they come without judging them as tough, and seeing all problems as challenges and tasks to be solved) they were not affected by high workload and stress.

The managers in this group mentioned their high resilience, positive personality, and active coping strategies. We also observed that some of the companies had several owner–managers, meaning managers’ tasks were shared by several persons.

#### c. Aware of the importance of sustainable working life from the beginning

Findings showed that managers in this group described that from the beginning they had had high awareness of the importance of sustainability at work and of maintaining a work–life balance. They intentionally strived to keep working hours to a moderate level, set clear boundaries between work and free time, and not work overtime. They described their health as stable and good and did not experience any change in wellbeing. Some managers mentioned that they had experienced work-related ill health, stress, and poor balance between their job and private life in previous jobs. They felt that this experience had helped them realize the importance of health. One manager learned from the example of his entrepreneur parents who had worked long hours. From the beginning, managers in this group had a high awareness of the importance of sustainable work life (and a high level of individual resources), which protected them from overworking and helped them maintain good levels of wellbeing.

#### d. Small companies with low organizational resources

The common feature of managers in this group is that their work situation was constrained by vulnerability characteristic of small businesses due to insufficient personnel and financial resources. These managers needed to work overtime to fill the personnel gaps and work operatively to earn their salary. They had to do administrative work, were unable to delegate tasks to others and could not invest time in the company’s development. These smaller companies also described some organizational adjustments; however, to a lesser extent. For example, they might hire a lower manager, or get help with finance, or with support systems, and developing improved routines. The managers also talked about trying to keep working hours at a moderate level. The managers in this group had low organizational resources but still felt well. Their working situation was constrained by the small size of their companies, but this did not translate into low wellbeing.

#### e. New in the manager role

This group consisted of managers who were new in their role of owner–manager or lower manager. Some had experienced heavy work demands when they had filled their role of manager, especially during the first period. After having problems with health these managers had acquired insight into the importance of wellbeing and had started to work intently on attaining and preserving a balance between work and life. They had also become more secure in their role after acquiring experience of working in a managerial position, and learned to delegate responsibilities to others, create better routines, prioritize actions, and not dwell too long on decisions. At the time of the interviews, the managers reported a clear improvement of their wellbeing compared with the first years of being in management.

Some of the newly promoted lower-level managers felt that their wellbeing had improved in their new position. They linked this to increased resources related to achieving larger responsibility, greater possibility to influence company development, more control over work and time, additional variation in work, and stimulating work.

## Discussion

The purpose of this study was to explore perceived changes in working conditions and wellbeing among managers of growing small businesses. To show how the results lead to conclusions regarding the purpose of the study, we first give a brief summary of the main findings and then discuss the observed changes in the managers’ wellbeing, their demands and resources, as well as changes in the context of small businesses itself in the process of growth. This is done by interpreting the findings and setting them in relation to the theoretical framework of the study.

The results indicate that managers’ working conditions in small companies evolve during periods of company growth. This leads to variations over time in managers’ experiences of wellbeing and work–life balance as well as to changes in job demands and resources. Managers’ working situation becomes less demanding and more manageable with a reduction in workload and working hours and a better work–life balance. The findings suggest that this perceived improvement may be due to changes in organizational factors, such as increased company resources, but also to managers’ personal insight based on their experiences, and to increasing awareness of the importance of a sustainable work situation. However, the analysis also showed that there were different trajectories in the way the perceived working conditions and wellbeing changed over time and how organizational and individual resources mattered for the managers’ wellbeing.

As mentioned previously, the basic assumption of the JD–R model is that specific job demands, and also resources, are rooted in specific occupational settings, i.e. they vary depending on the work settings and the context of the organization [27]. The present study, building on the JD–R model’s assumptions, shows further that the specific context of small companies is itself subject to changes when a company expands and evolves. In other words, the results of this study illustrate that change occurs in a company over time because of the growth, which refers to an aspect of dynamism that occurs in the small business context. Changes in managers’ wellbeing, job demands, and resources in the context of small business growth are explicated below.

Concerning wellbeing, previous research reported good health and job satisfaction with regard to both managers [45, 48] and entrepreneurs [9, 14, 69–71], although some few studies showed the opposite (e.g. [49, 51, 53, 54]). This study provides a more nuanced understanding of managers’ wellbeing in the context of small businesses. Like previous research, the findings in this study point out that managers felt well and experienced job satisfaction and good work–life balance despite the high demands they faced. Although they felt well at the time of the interview, many owner–managers had also experienced impaired wellbeing in previous periods when their company had been smaller and weaker, as shown in the description of the first trajectory group. Thus, the findings suggest that owner–managers in small businesses risk impaired wellbeing due to high workload, long working hours, and work–life conflict when the company is particularly small and when managers lead the growing company mostly by themselves. Also, new managers at low and higher levels, as demonstrated by trajectory group 5, seem to be at risk of diminished wellbeing due to increased job demands, especially during the first years of their managerial career. Increased demands due to transition to a managerial position have also been shown in previous studies [47, 72].

Moreover, our results indicate that companies’ increased resources due to growth had implications for managers’ working conditions and wellbeing. First, the managers’ workload decreased because of increased possibilities to delegate a part of their tasks to lower-level managers and because of the increased number of personnel. Second, larger resources, better organization and routines reduced the degree of uncertainty and increased the preparedness and capacity to tackle arising problems, and thus increased the sense of manageability and reduced the intensity of the demands. The study shows that the decreased demands and increased organizational resources led to improved wellbeing for managers, as illustrated in the first trajectory group. Therefore, growth may have a positive effect on managers’ working conditions, primarily for higher managers in small growing companies. However, results from the study also indicate that growth can itself be a stressor, requiring constant adjustments and changes in the organization. If not well handled, growth can result in problems and tensions. Company growth, therefore, creates a changed situation that requires new strategies, new ways of working, and adjustments in an organization.

In relation to the organizational context, the present study distinctly points to the changing nature of the organization undergoing growth. More specifically, the study suggests that, during the process of growth, there is an increase in the degree to which an owner delegates their responsibilities as well as in the complexity of organizational structure (such as management levels) and operational systems (such as financial and production management systems). There also is a decrease in an owner’s involvement in business activities and daily decisions. Increased labour specialization, formalization, standardization (e.g. work with routines), planning and control as well as reduced proximity in relations with employees were some of the transformations that companies went through. Transformations may mean changes in the content of managers’ work, demands (e.g. decreased demands related to managers’ daily work, lower involvement in operational activities and lower working hours) and resources (e.g. in the form of a larger staff, personnel with special competence, higher use of operational systems, formalization and routines, and greater financial security due to larger resources). The described transformations could be traced to all the companies in the study that were growing, and thus represent a background characteristic of all the trajectory groups except for the fourth group (companies that did not continue to grow). The changes are generally in line with the transformations described in Churchill and Lewis’ [62] model, but also with Torrès and Julien’s [64] discussion on the denaturing of small business, as presented above.

According to the findings in the present study, even the features of small companies that give specificity to the management modes in this context are subject to change when a company expands. Applying Torrès and Julien’s [64] view, the current findings may indicate that companies in the process of growth “denature” and lose their small businesses specificity. Thus, businesses transition from simpler, more intuitive, and informal approaches to management, which are characterized by close relationships, to more complex, structured, and formalized modes that focus on long-term planning and less personal interaction [64]. This may even apply to managers’ work. As mentioned previously, managers in the smallest of small companies have a special position combining the managerial roles of several different levels: being the owner, the entrepreneur, the operative worker, the administrator, etc. (referring to the fourth trajectory group and the initial situation for the first, second and third groups). When a company expands the owner–manager’s work and role transform and become more like those of managers in larger companies (as described for the first trajectory group). The findings thus point to the special working conditions of owner–managers of small companies (characterized by a combination of different roles, resource constraints and the changing nature of their work in the process of business growth) while middle managers’ working conditions and wellbeing in these companies are more in line with what previous research has shown about managers in general.

In the current study, the smallest companies were vulnerable because of poor financial and personnel resources, while the larger small companies did not experience this vulnerability. The growing companies were able to enhance their personnel, financial and organizational resources thanks to growth, which allowed them to overcome the vulnerability related to small business size. This means that these companies built up a stronger reserve pool, which led to higher resilience, allowing them to endure acute and chronic stressors, prevent resource loss and ensure future resource gain [73–75]. The companies in the study that continued to grow seemed to have a resource surplus; and developed in positive spirals in relation to economic growth as they continued to grow steadily. Having a resource surplus or strong resource reservoirs can obviously be a protection and resource factor for managers’ wellbeing.

Interestingly, the results showed that managers in the smallest companies (companies that had had a short period of growth and did not continue to grow, as shown in the fourth trajectory group) experienced good wellbeing despite high demands. Two possible explanations might help understand these findings. First, as described above, it seems that the available personal resources had a protective effect. Second, it is possible that these companies may have attained the size and mode of operation that allowed a manageable working situation for managers. These companies experienced small business vulnerability due to low resources but remained stable. They were able to engage in reactive coping with daily stressors (e.g. sickness among staff, or machine breakdowns) and handle the situation and keep the balance, even though they were currently not able to invest in growth. Their lack of resources did not seem to lead to negative spirals; however, vulnerability remained. In other words, in case the external environment changes, e.g. in an economic recession, they may be at risk of escalating resource depletion.

An interesting finding of the study is that managers in general seemed satisfied with their job despite high workload both previously and currently. In relation to owner–managers, an entrepreneurial dimension in their job should be noted. Entrepreneurs’ work is self-chosen and the workload is quite often self-inflicted as well. Having a lot of work and solving problems can be the source of motivation, wellbeing, and work satisfaction for an entrepreneur. Entrepreneurs choose to have a lot of work and see this as a sign that everything is going well for the business. At the same time, demands related to high workload and pace may lead to lower wellbeing in the long term [14]. There seemed to be dual experiences of workload in the owner–managers’ work.

Finally, the study indicates that individual resources may affect managers’ working conditions. Firstly, these relate to managers’ awareness of the importance of health for their own and their companies’ sustainable working life. Secondly, the findings showed the significance of acquiring managerial experience as well as learning the own profession, and the work content and specific situation in the company.

It should be noted that we observed an increase in organizational resources in all growing companies and the participants from these companies admitted that an increase in organizational resources had improved their working conditions and reduced their workload. However, it seems that this had the most pronounced effect on improvement of wellbeing of owner–managers in the first trajectory group. It appears that the first group differed from the other groups of managers in growing companies (the second and third trajectory groups] in the way that they initially lacked individual resources in terms of awareness of the importance of wellbeing and sustainable working life. Those managers who initially enjoyed large individual resources did not overwork and therefore did not experience deterioration in wellbeing. This may suggest that individual resources can have a protective effect [16]. The findings further show that several managers developed a greater understanding of the importance of sustainable working life after having had problems with wellbeing and work–life balance (e.g. in the first and third trajectory groups). Thanks to this increased understanding, managers changed their behaviours (e.g. by keeping working hours to a moderate level or taking some time off after a period of hard work), which contributed to a reduction in their workload, which in turn had a positive impact on their wellbeing. Therefore, the study’s results suggest that there is feedback from managers’ wellbeing to their personal resources. Before concluding the paper, an outline of the study’s limitations and the practical implications of the findings are highlighted in the section below.

### Theoretical and practical implications

This study responds to calls to deepen the understanding of occupational health of small business managers [76, 77], to pay more attention to variability and temporal aspects in the work and wellbeing of small business managers [8, 14].

The main contribution of this study is that it brings attention to the dynamic, fluid and contextually conditioned nature of managers’ work in small growing companies, and its implications for their wellbeing, as well as the interconnectedness of managers’ work, work organization and wellbeing. This clearly adds to previous research, largely offering a static view of managers’ wellbeing. Additionally, the study employed an interdisciplinary approach, integrating theoretical perspectives and empirical research from research areas of occupational health, management studies, business growth, and entrepreneurship. This and usage of qualitative approach contributes to a deeper and more nuanced understanding of managers’ working conditions and wellbeing in the particularly under-researched context of small growing firms, adding to the previous research characterised by predominantly quantitative approaches, and largely confined to a single research domain.

In terms of practical implications, the study’s results can support leaders in maintaining their own health when running small businesses and pursuing growth and economic effectiveness of the company. Thus, small business managers, particularly at the beginning of their careers, would benefit from developing an awareness of the role of wellbeing for their work and their organization. The study also delineates sources of occupational stress that may be detrimental to their wellbeing and available resources that may help to support and strengthen their wellbeing. Thus, the study draws attention to the importance of promoting a healthy work environment for both owner-managers and lower managers in small businesses. Managers should also be aware that high workload and long working hours constitute a risk to their wellbeing with potentially negative consequences for their companies. Information about factors important for the wellbeing of small business managers can be used in training programs for this group. Also, managers should be coached to participate in various professional peer networks to discuss their working situation, receive support and shared experience, learn how to create clearly defined boundaries for when they are working and not working to ensuring that they do not overwork.

The study may also inspire relevant stakeholders such as politicians, trade unions of employers and other decision makers to develop appropriate and feasible ways and structures (e.g. education kits for entrepreneurs, mentorship, shared resource pools for administrative work and human resources management etc. for several businesses) to reduce the sensitivity that start-ups and small businesses live with, to increase managers’ and companies’ resources, improving managers’ working conditions and therefore their wellbeing.

### Limitations and future research

Altogether, it seems that the different pathways described in the trajectories led to higher resilience and a more sustainable working situation for managers thanks to reduced demands and increased resources. However, we are aware that the study sample, consisting of growing successful companies having survived for several years in a row, may have implications for our conclusions as companies that had not survived and where managers’ wellbeing may have led to entrepreneurial exit (i.e. when an owner–manager leaves or closes the firm) were not included. Previous research has indicated that most small businesses do not survive their first years of operation [78] and owner–managers’ wellbeing is associated with their exit intentions [79]. Future research should explore, qualitatively and quantitatively, the cases where managers’ wellbeing status led to entrepreneurial exit.

Another question that should be addressed in future studies is whether growth initially demands extra investment of resources from managers to ensure continuing growth. When managers lack the necessary resources (which is the case in many small businesses) they often need to work extra to save costs, or earn more to create the necessary surplus to ensure growth.

Assumingly, if the sample had had an even distribution of gender the results may have looked different, for instance in relation to work–life balance as women often do not have the same possibility to work extremely long hours as the men in our study did.

A possible limitation of the study is that the categories *currently* and *previously* may differ between individual managers. *Currently* could imply today but could also cover the last few years. Similarly, *previously* could mean last year but it could also mean 10 years ago. These discrepancies are due to the fact that the companies were in different stages of growth and managers had varied length of managerial experience. This has implications for the granularity of the trajectory analysis.

Furthermore, although the study results indicated changes in perceived wellbeing over time, these findings need to be interpreted with caution because of the small sample size. Additionally, the study relied on a qualitative design. Therefore, future research is warranted using other methodologies (e.g. quantitative). The trajectory analysis did not aim to identify general patterns in managers’ evolving wellbeing, demands and resources in relation to small companies’ growth; it merely was an attempt to illustrate that participants perceived those changes occurred in different ways because of an interplay between organizational and individual resources.

Finally, this study relied on managers’ subjective experiences and perceptions of their working conditions and wellbeing, which they felt reflected their current situation. Nevertheless, it is important to be aware of other perspectives which could see managers’ narratives as socially constructed. The findings of the study, for instance, show the importance of managers’ individual resources. This could be discussed in relation to the view of managers as either doers or heroes in the research streams that oppose exaggerating managers’ role in a company’s success and failures [80]. It could be argued that what managers share regarding their experiences and perceptions can be seen as an expression of their socially constructed identity of strong and action-oriented entrepreneurs whose actions are decisive for business success; and that they perhaps overemphasize their own individual contribution. Therefore, research capturing these experiences using other methods such as observations or discourse analysis is warranted.

Also, it can be assumed that those who felt satisfied with their job, wanted to share their success story, and had more time were more inclined to take part in the study. Those who could barely keep their heads above water may have been more likely to decline participation – both because of stress and because they could not live up to the narrative.

## Conclusion

This study shows the dynamic picture of small business managers’ working conditions and wellbeing that is due to the growth-related changes in the company and the managers’ work. Managers’ experiences of own wellbeing, the posed demands, and available resources changed over time in the process of the companies’ growth. When the companies were small, there was a risk for impaired wellbeing among owner–managers because of high workload, long working hours, and work–life imbalance. In addition, the study shows a positive impact of increased organizational resources brought through the company’s growth, leading to reduced workload, improved wellbeing, and work–life balance for managers. Furthermore, the perceived improvements were due not only to the changes in organizational factors, but also to managers’ personal insights and an increased awareness of the importance of a sustainable work situation. Finally, the results showed that the perceived changes in managers’ working conditions and wellbeing followed different trajectories over time because of the interaction between organizational and personal factors.

## Data Availability

The data presented in the study are available on reasonable request from the corresponding author. The data are not publicly available owing to restrictions in the ethical approval of this study.
